# Individual differences in leech heart motor neuron models

**DOI:** 10.1186/1471-2202-13-S1-O9

**Published:** 2012-07-16

**Authors:** Damon G Lamb, Ronald L Calabrese

**Affiliations:** 1 Department of Biology, Emory University, Atlanta, GA 30322, USA

## 

Motor neurons are frequently overlooked as critical contributors to the programming of motor output except where they directly play a role in central pattern generators (CPGs). Leech heart motor neurons have been shown to contribute significant phase shifts to the rhythmic motor patterns they produce [1-3], although the most important factor in pattern formation is the interaction of CPG output and synaptic weights from the CPG onto the motor neurons [3]. We seek to address the question of which neural parameters, in particular active conductances, are important for functional pattern formation, and how they influence it. We are well positioned to address this question, as we have a unique dataset comprising the complete leech heart motor neuron input pattern, output pattern, and synaptic weights for multiple ganglia in many individual animals. We have begun to exploit these data to develop more realistic Hodgkin-Huxley style biophysical models of the leech heart motor neurons by constraining models optimized by a multi-objective evolutionary algorithm to fit this animal-specific data. In particular, we extended the single compartment Garcia model [1] to a multi-compartmental model and added a slow calcium and a calcium sensitive potassium channel, currents known to be present in the living motor neurons. Our model’s dimensions are derived from reconstructions of fluorophore filled leech heart motor neurons in segments 8-12. Membrane currents were distributed according to experimental data and the result of hand tuning; e.g., the fast sodium current is only present in the axonal compartment. This base model was then parameterized such that the maximal conductances of the active currents present in each compartment, as well as the electrical coupling conductance between each pair of motor neurons, were allowed to vary as free parameters in our evolutionary algorithm. We then utilized our input-output data by delivering a particular input pattern (specific animal and motor neuron pair) to a model motor neuron and then comparing the resulting output with that recorded in the same animal. In this manner, we constrained our definition of an ‘acceptable’ model by the input/output pattern recorded in individual animals – such a model must transform the input into the correct output within some reasonable error bound. To generate acceptable models we use a Multi Objective Evolutionary Algorithm (MOEA) to create a population of models from which a subset achieves ‘acceptable’ status. In this framework, multiple independent fitness functions are evaluated separately – the phase, duty cycle, spike height, slow-wave height, and within-burst inter-spike interval (ISI) for the two phases produced. The targets for these parameters are drawn from the same individual animal as the input/output data, and error bounds are based on the within-animal variability, although slightly widened for parameters such as ISI, slow-wave and spike height, as they vary greatly between animals (derived primarily from within-animal experimental variability). Acceptable model simulation traces were all but indistinguishable from intracellular recordings from the living system, with the exception of measurement noise, even though they are constrained by a small number of functional parameters. In order to identify key parameters and relationships between synaptic weights and membrane conductances which are constrained by the functional requirements of the system, we will conduct a sensitivity analysis of the acceptable models and inspect the dimensional stacking [4] of acceptable and failed models. The identified conductances can then be perturbed in the living system either via dynamic clamp or pharmacological manipulation to validate the modeling results.

## References

[B1] Garcia PS Wright TM Cunningham IR Calabrese RL Using a model to assess the role of the spatiotemporal pattern of inhibitory input and intrasegmental electrical coupling in the intersegmental and side-to-side coordination of motor neurons by the leech heartbeat central pattern generator J Neurophysiol 2008 100 3 1354 1371 10.1152/jn.90579.2008 18579654 PMC2544471

[B2] Wright TM Jr. Calabrese RL Contribution of motoneuron intrinsic properties to fictive motor pattern generation J Neurophysiol 2011 106 2 538 553 10.1152/jn.00101.2011 21562194 PMC3154826

[B3] Wright TM Jr. Calabrese RL Patterns of presynaptic activity and synaptic strength interact to produce motor output J Neurosci 2011 31 48 17555 17571 10.1523/JNEUROSCI.4723-11.2011 22131417 PMC4127079

[B4] Langton JT Gifford EA Hickey TJ Visualization and Interactive Exploration of Large, Multidimensional Data Sets Stud Comput Intell 2008 122 231 255 10.1007/978-3-540-78534-7_10

